# Supplementary material for: The genetic architecture of sporadic and multiple consecutive miscarriage
Source: Nat Commun. 2020 Nov 25;11:5980. doi: 10.1038/s41467-020-19742-5 (PMC7689465; doi:10.1038/s41467-020-19742-5)
Supplement: Supplementary file 1 — Supplementary Information [file 41467_2020_19742_MOESM1_ESM.pdf]

Supplementary Information

The genetic architecture of sporadic and multiple consecutive miscarriage

Laisk et al.

Supplementary Methods

Description of participating cohorts

UKBB

The UK Biobank (UKBB) is a prospective cohort of 502,637 (~5% of the >9.2 million invited) people aged 37-73 recruited in 2006-2010 from across the UK, who completed detailed questionnaires regarding socio-demographic and lifestyle characteristics and their medical history, and had a clinical assessment. Additional information about medical conditions (both existing at baseline and occurring during follow-up) has been obtained through linking with hospital admission and mortality data. Full details of the study have been reported elsewhere [1]. Ethics approval for the UKBB was provided by the UK National Health Service (NHS) Research Ethics Service (11/NW/0382) and all participants provided informed written consent.

Information on miscarriages was retrieved from touchscreen questionnaire field 3839 "How many spontaneous miscarriages?", which is collected from participants who indicated they had had a stillbirth, spontaneous miscarriage or termination, as defined by their answers to data field 2774 "Have you ever had any stillbirths, spontaneous miscarriages or terminations?". We additionally used data from hospital in-patient episode data fields 41202 and 41204 ("Diagnoses – main ICD10" and "Diagnoses – secondary ICD10", diagnosis codes O02.1 and O03, and N96). In the UKBB, most of the data has been collected during the initial assessment visit, however, for some participants, data have been additionally collected on repeat assessment visits. Therefore, if a participant has answered the same question on multiple occasions, answers were aggregated, excluding participants who have given discordant answers on different occasions. Participants giving no answer to relevant questions, or answering "Prefer not to answer" or "Do

not remember" were excluded from the analysis. Analyses were performed under data applications 17805 ("Dissemination of shared genetics across phenotypes associated with reproductive health and related endophenotypes"), 11867 ("Dissection of the Genetic Susceptibility of Obesity Traits and their Comorbidities"), and 16729 ("MR-PheWAS: hypothesis prioritization among potential causal effects of body mass index on many outcomes, using Mendelian randomization"). The GWAS analysis included 37,150 women of White European ancestry with self-reported or electronic health record-derived sporadic miscarriage, 421 with multiple consecutive miscarriage and 164,775 female controls (no miscarriages and no exclusion diagnoses) with available genome-wide data. The sporadic miscarriage trans-ethnic meta-analysis also included 511 cases and 1,424 controls of UK South-Asian ancestry, 390 cases and 957 controls of UK Caribbean ancestry, 132 cases and 433 controls of UK Chinese ancestry, and 273 cases and 482 controls of UK African ancestry.

EGCUT

The Estonian Genome Center of the University of Tartu (EGCUT; http://www.biobank.ee) is a population-based biobank with a total cohort size of 51,515 participants (aged 18-85+)[2]. The EGCUT cohort included a total of 3,368 women of White European ancestry with sporadic miscarriage, 113 with multiple consecutive miscarriage, and 17,996 women as controls. Information on miscarriages was retrieved from questionnaire fields "How many times have you got pregnant?" and "How many of the pregnancies ended with unintentional miscarriage?", and based on ICD codes obtained from linking with Health Insurance Fund databases. The study was approved by the Ethics Review Committee of the University of Tartu (234T-12). All biobank participants have signed a broad informed consent form.

ALSPAC

The Avon Longitudinal Study of Parents and Children (ALSPAC) is a prospective pregnancy/birth cohort that recruited 14,541 pregnancies of women resident in Avon, UK with expected dates of delivery 1st April 1991 to 31st December 1992. These women delivered 14,062 live births and they, their partners and offspring have been followed-up since then with detailed repeat questionnaires, hands-on clinic assessments and record linkage. Full details of the study can be found elsewhere [3]. ALSPAC is an accessible resource for the research community and the study website contains details of all the data that are available through a fully searchable data dictionary (http://www.bris. ac.uk/alspac/researchers/data-access/data-dictionary). Ethical approval for the ALSPAC was obtained from the ALSPAC Ethics and Law Committee and the UK National Health Service Research Ethics Committee (full details at http://www.bristol.ac.uk/alspac/researchers/data-access/ethics/lrec-approvals/#d.en.164120). Women provided written informed consent.

At recruitment and follow-up, the women have been asked about miscarriage and stillbirth. For this study, we considered miscarriages reported at baseline regarding pregnancies before the index child, and at subsequent follow-ups regarding pregnancies after the index child. At baseline, women were asked “Have you ever had any miscarriages?” and “How many times have you miscarried?”. At the follow-up assessments, miscarriage was retrieved from information asked about: i. outcome of each pregnancy after the index child (i.e. “Since child’s <age at follow-up>’s birthday, have you become pregnant?”, “What happened in the 1st pregnancy?”, “What happened in the 2nd pregnancy?”), ii. occurrence of miscarriage (i.e. “Since the study child was <age at follow-up>, have you had a miscarriage?”), and iii. whether a dilation and

curettage (D&C) occurred due to miscarriage (i.e. "Have you had a D and C (scrape) in the last 2 years?" "Was this because of miscarriage?"). In total, 1,473 women had sporadic miscarriage, 216 had multiple consecutive miscarriage, and 4,475 women had no miscarriage.

QIMR

The QIMR samples were drawn from two cohorts of adult twins and their relatives (parents, siblings, adult children and spouses) who have taken part in a wide range of studies of health and well-being via previous postal questionnaires and telephone interview studies (questionnaires and methods are summarized in Medland et al., 2008 [4]). As a result, this sample includes related individuals, 1,145 women reporting sporadic miscarriage and 5,136 women who did not report experiencing miscarriage as controls. The QIMR Endo samples were drawn from a cohort of women with a confirmed surgical diagnosis of endometriosis, and for whom detailed reproductive history data are available [5,6]. This sample includes only unrelated individuals, 497 women reporting sporadic miscarriage and 1,078 women who did not report miscarriage as controls. For both samples miscarriage information was drawn from questionnaire fields "Have you ever had a miscarriage?" and "Number of miscarriages?". Ethics approval for studies involving these individuals was granted by the QIMR Human Research Ethics Committee. Informed consent was obtained from all participants.

iPSYCH

The iPSYCH2012 (Lundbeck Foundation Initiative for Integrative Psychiatric Research) dataset represents a Danish case-control cohort (a total of 76,657 participants) for psychiatric disorders. The iPSYCH cohort included a total of 1,173 women with sporadic miscarriage and

4,821 women as controls (all of White European ancestry). Women were considered sporadic miscarriage cases if they had an inclusion code (O02.1 and/or O03) on one or two separate occasions in their medical record. At the same time, individuals with any of the exclusion diagnoses mentioned below were not included in the analysis. Controls were selected from women who had not had a miscarriage and were matched based on genotyping wave, four controls for each case. The iPSYCH GWAS data were generated based on dried blood spot samples obtained during routine neonatal screening and stored in the Danish Neonatal Screening Biobank. Parents are informed in writing about the neonatal screening and that the samples can later be used for research, pending approval from relevant authorities[7]. The study was conducted under the iPSYCH study ethics approval given by the Danish Research Ethics Committee and has also been approved by the Danish Data Protection Agency.

Lifelines

The Lifelines dataset included in this study represents a subset of samples with available genotype data from the Lifelines prospective population-based cohort [8,9], examining in a unique three-generation design the health and health-related behaviours of 167,729 persons living in the North of The Netherlands. It employs a broad range of investigative procedures in assessing the biomedical, socio-demographic, behavioural, physical and psychological factors which contribute to the health and disease of the general population, with a special focus on multi-morbidity and complex genetics. The Lifelines dataset was accessed under data application OV17-0393. Self-reported information on miscarriages was extracted from questionnaire field "How many miscarriages (up to 16 week) have you had?". The sample includes 1,676 sporadic miscarriage cases and 5,091 female controls. The Lifelines study has been approved by the

review board of the University Medical Center, Groningen, and adheres to the principles expressed in the Declaration of Helsinki. All study participants provided written informed consent.

Partners HealthCare Biobank

The Partners HealthCare Biobank is a biorepository of consented patient samples at Partners HealthCare (the parent organization of Massachusetts General Hospital and Brigham and Women's Hospital). All patients who participate in the Partners Biobank are consented for their samples to be linked to their clinical information for researchers at Partners HealthCare to conduct research on human health (including genomics, epidemiology and outcomes research) for which institutional review board approval was obtained. For the analyses described in this paper, only European American patients were included due to sample size. Sporadic miscarriage cases were identified using ICD codes and the same inclusion and exclusion criteria described above, resulting in 58 cases and 289 controls for analysis.

MoBa-HARVEST

The Norwegian Mother and Child Cohort Study (MoBa) is a prospective population-based pregnancy cohort study conducted by the Norwegian Institute of Public Health. Participants were recruited from all over Norway from 1999-2008. The women consented to participation in 41% of the pregnancies. The cohort now includes 114 500 children, 95 200 mothers and 75 200 fathers. Blood samples were obtained from both parents during pregnancy and from mothers and children (umbilical cord) at birth. The cohort is described in the following publications [10–12]. In the current study, a subset (n=8,000) of the MoBa cohort (genotyping effort HARVEST, also

known as Njolstad1) is included. Sporadic miscarriage cases were defined based on questionnaire-derived data, and controls were also restricted to have at least one previous delivery. The analysis included 1,653 sporadic miscarriage cases and 3,199 female controls.

A signed informed consent was obtained from all study participants. The administrative board of the Norwegian Mother, Father, and Child Cohort Study led by the Norwegian Institute of Public Health approved the study protocol. The establishment of MoBa and initial data collection was based on a license from the Norwegian Data Protection Agency and approval from The Regional Committee for Medical Research Ethics. The MoBa cohort is currently regulated by the Norwegian Health Registry Act. This study was approved by the Regional Committee for Medical and Health Research Ethics from Norway (2015/ 2425).

BGI Chinese millionome database Phase I 140K study

The BGI cohort represents a dataset of 141,431 Chinese women generated for non-invasive prenatal testing (NIPT), for whom whole-genome low-pass sequencing data are available [13]. The study was reviewed and approved by the Institutional Review Board of BGI (BGI-IRB17088) in strict compliance with regulations regarding ethical considerations and personal data protection. A signed informed consent was obtained from all study participants. A subset of 135,115 participants who reported their pregnancy history were included in this study, resulting in 8,865 sporadic miscarriage cases and 126,290 controls for analysis.

China Kadoorie Biobank

The China Kadoorie Biobank is a prospective population-based cohort of 512,891 adults aged 30-79 years recruited from 10 geographically defined regions during 2004-2008, with

collection of questionnaire data, physical measurements and blood samples [14]. The current study included data for 57,622 women, from whom 5,038 sporadic miscarriage cases and 51,696 controls who had ever been pregnant were extracted based on self-reported data on miscarriages. Ethical approval for China Kadoorie Biobank was obtained jointly from the University of Oxford, the Chinese Center for Disease Control and Prevention (CCDC), and the regional CCDC from the 10 study areas, and all participants provided written informed consent.

Women's Health Initiative

The Women's Health Initiative (WHI), sponsored by the National Heart, Lung, and Blood Institute (NHLBI), is a long-term national health study that focuses on strategies for preventing heart disease, breast and colorectal cancer, and osteoporosis in postmenopausal women. Our meta-analysis included data from several GWAS substudies from the WHI project: WHI HIPFX (mostly European ancestry case-control study for hip fracture; 273 sporadic miscarriage cases and 708 controls), WHI Gecco cyto (European ancestry case-control study by the Genetics and Epidemiology of Colorectal Cancer Consortium; 274 sporadic miscarriage cases and 609 controls), WHI WHIMS (WHI Memory Study; 411 sporadic miscarriage European ancestry cases and 950 controls), WHI GARNET (Genomics and Randomized Trials Network cohort study; 954 sporadic miscarriage cases and 2,096 controls of European American ancestry), WHI SHARE (SNP Health Association Resource; 1,151 sporadic miscarriage cases and 2,195 controls of Hispanic American ancestry, and 2,919 sporadic miscarriage cases and 4,546 controls of African American ancestry). Cases were identified based on self-administered questionnaire data from variable phv00078626.v6.p3 ('How many miscarriages or unspecified type of pregnancies').

The human genetic analysis of the WHI cohorts reported in this study were reviewed and approved by the Institutional Review Board of Washington University in St. Louis, under protocol number #201109261 and all participants provided informed consent.

Gene-based analyses

Gene-based genome-wide association analysis was carried out with MAGMA 1.06 [15] (Multi-marker Analysis of GenoMic Annotation) with default settings implemented in FUMA [16]. Briefly, variants located in the gene body were assigned to respective protein-coding genes (n=18,929; Ensembl build 85), and the resulting SNP *P*-values are combined into a gene test-statistic using the SNP-wise mean model [15]. To adjust for multiple testing, genome-wide significance level was set at 2.6×10^{-6}, according to the number of tested genes. No genes passed the threshold of significance.

Look-up in GWAS browsers

In order to assess the potential genetic overlap between miscarriage phenotypes and other traits, we conducted a lookup in the publicly available GWAS catalogue (e91_r2018-02-06) (https://www.ebi.ac.uk/gwas/), and GWAS browsers Oxford BIG browser (http://big.stats.ox.ac.uk/) and GWASAtlas [17] (http://atlas.ctglab.nl/) for the miscarriage-associated variants identified in any of our three GWAS meta-analyses. One of our variants associated with multiple consecutive miscarriage (chr 9: rs7859844 showed nominal association with 'stomach or abdominal pain' (P=4.9×10^{-6}) and venous thromboembolic disease (P=$5.8\times10^{-}$

$^{6})$[17], 'CD314-CD158a+ NK cell proportion' ($P=3.3\times10^{-6}$) and CD32+ mDC (dendritic cell) subset proportion ($P=8.7\times10^{-6}$)[18].

Functional annotation with FUMA (v1.3.1)

As the first step, independent significant SNPs in the GWAS meta-analysis summary statistics were identified based on their P-values ($P<5\times10^{-8}$) and independence from each other (r2<0.6 in the 1000G phase 3 reference) within a 1Mb window. Thereafter, lead SNPs were identified from independent significant SNPs, which are independent of each other (r2<0.1). SNPs that were in LD with the identified independent SNPs (r2≥0.6) within a 1Mb window, have a MAF of ≥1% and GWAS meta-analysis *P*-value of >0.05 were selected as candidate SNPs and taken forward for further annotation.

FUMA annotates candidate SNPs in genomic risk loci based on functional consequences on genes using the Annotate Variation (ANNOVAR, 2017-07-17) [19], CADD (a continuous score showing how deleterious the SNP is to protein structure/function; scores >12.37 indicate potential pathogenicity) [20] and RegulomeDB [21] scores (ranging from 1 to 7, where lower score indicates greater evidence for having regulatory function), 15 chromatin states from the Roadmap Epigenomics Project [22,23], eQTL data (GTEx v6 and v7) [24], blood eQTL browser [25], BIOS QTL browser [26], BRAINEAC [27], MuTHER [28], xQTLServer [29], the CommonMind Consortium [30], and 3D chromatin interactions from HI-C experiments of 21 tissues/cell types [31]. FUMA maps genes to candidate SNPs using positional mapping, which is based on ANNOVAR annotations and maximum distance between SNPs (default 10 kb) and genes, eQTL mapping and chromatin interaction mapping. Chromatin interaction mapping was performed with significant chromatin interactions (defined as $FDR<1\times10^{-6}$). The two ends of significant chromatin

interactions were defined as follows: region 1, a region overlapping with one of the candidate SNPs; and region 2, another end of the significant interaction, used to map to genes based on overlap with a promoter region (250 bp upstream and 50 bp downstream of the transcription start site).

MR-PheWAS

The nature of PheWAS is that they are hypothesis generating. Whilst the application of a stringent p-value threshold helps to minimise false positive results, any suggestive associations need replication in independent datasets and appropriate sensitivity analyses to explore whether the associations are likely to be causal or reflect horizontal pleiotropy. This approach is not able to determine conclusively the nature of any causal effect, thus outcomes that are statistically significantly related to the exposure of interest (here multiple consecutive miscarriage) might be confounded. In this specific example, the MR-PheWAS was undertaken in a study that is not independent of the GWAS sample that identified the multiple consecutive miscarriage hits (it is the largest study contributing to that GWAS) and there may be over-fitting of the data. Furthermore, with only four rare SNPs the use of MR-Egger to explore horizontal pleiotropy may be unreliable. Lastly, the proportion of variation in multiple consecutive miscarriage explained by the genetic risk score (between 0.02% to 0.6%) is low (McFadden's adjusted R2=0.0006, Efron's R2=0.0002, Pseudo R2=0.0062), suggesting that we might have weak instrument bias. Given these considerations and the fact that the three associations that reached significance below our predefined Bonferroni threshold did not seem plausible causes or consequences of multiple consecutive miscarriage we did not explore any of those further. We did look at other outcomes in the top 10 lowest *P*-values (i.e. the group that deviate from the QQ

plot; Supplementary Figure 2) to see if any were more plausible. Supplementary Table 10 lists the top 10 (lowest *P*-values) outcomes with per multiple consecutive miscarriage allele association (SD) and *P*-value. With one exception none of these are outcomes that have been shown in previously published, or our, multivariable regression analyses and do not seem biologically plausible. The one exception is a suggestive causal effect of multiple consecutive miscarriage on endometriosis of the uterus (P=5.9×10^{-5}).

Two-sample Mendelian randomization of endometriosis on multiple consecutive miscarriage

We used a two-sample MR approach [32] to explore the possible causal effect of endometriosis on multiple consecutive miscarriage. We obtained summary association results for genetic instruments for endometriosis from a recent meta-analysis of 11 genome-wide association case-control data sets[33]. Summary associations between each instrument and multiple consecutive miscarriage were estimated using Firth regression in the three European ancestry studies of our GWAS and meta-analysed using METAL [34]. From the 19 SNPs associated with endometriosis, 18 were present in at least two of our three studies. After harmonization of the summary data sets, we used inverse variance weighting (IVW) [35] and MR-Egger [36] to obtain a pooled estimate of the association between the SNPs for endometriosis and multiple consecutive miscarriage. There was no evidence of a causal association between endometriosis and multiple consecutive miscarriage (IVW OR 1.18, 95% CI 0.84, 1.65 and MR-Egger OR 0.96, 95% CI 0.23, 4.02) (Supplementary Figure 9).

Colocalization analyses of multiple consecutive miscarriage and expression/protein levels

Associations between SNPs and protein levels were obtained from the INTERVAL study, where 2,994 unique proteins (3,283 measured aptamers) had been investigated in up to 3,301 participants of European origin [37]. We extracted genotype-expression data from the GTEx project version 7, with cis-genotype-expression data available for 48 different tissues [38]. The sample sizes in GTEx varied between 80-491 depending on tissue, and approximately 85% of donors were of European origin (denoted "White" by GTEx) [38]. We excluded one SNP (rs144887114) for the transcripts *TLE1* and *RP11-154D17.1* in the terminal ileum expression dataset because of unlikely effect estimates (over a trillion). Lastly, we used data from a recent cis-eQTL study in placenta, including 40 samples of European ancestry [39]. For the placental data, we matched the rsIDs to genome position using GRCh 37 and the biomaRt R package [40,41]. We further matched genome position to rsIDs using the file "All_20180423.vcf.gz" available at ftp://ftp.ncbi.nih.gov/snp/organisms/human_9606_b151_GRCh37p13/VCF/ [42] for multiple consecutive miscarriage. In all datasets, we removed SNPs that were missing relevant data, duplicated by position, or indels.

We constructed an LD-reference panel using a subset of the UK Biobank [1] and the imputed data for use in the GCTA analyses. Briefly, we extracted a random subsample of 27,625 participants in the "White British ancestry" subset, after excluding individuals that had withdrawn consent, mismatch between self-reported and genetically inferred sex, sex-chromosome aneuploidy, reported incompatible ancestries in different assessments, or that were related to other individuals in the UK Biobank to a third degree or higher, heterozygosity or missingness outliers, or not included in the autosome phasing or in the kinship calculations [43]. Genotype dosages were converted to best-guess genotypes using a hard-call threshold of 0.1. We excluded SNPs with an imputation info score ≤0.3, minor allele frequency <0.01%, Hardy-

Weinberg equilibrium exact test $P<1\times10^{-6}$, or genotype missing call rate >0.05. Analyses were done in plink versions 1.90b3 and 2.00a-20170724 [44].

To compute estimates conditioned on the top expression SNPs, we used GCTA version 1.91.4 [45,46]. Information on effect allele frequency for GTEx were taken from the files "GTEx_V7_cis_eqtl_summary.tar.gz (hg19)" (downloadable at http://cnsgenomics.com/software/smr/#DataResource). We first selected the top SNP using GCTA (option --cojo-top-SNPs), and thereafter computed estimates conditioned on the top SNP (option --cojo-cond).

Colocalization analyses were performed using R versions 3.4.3 and 3.5.1 (https://www.r-project.org/), the coloc R package [47] and the coloc.abf() function using two different region sizes: 2 Mb (or all available SNPs depending on dataset), as well as 400 kb as a sensitivity analysis. In INTERVAL, we investigated all proteins consisting of a gene with a transcription start site within 5 Mb of a top miscarriage SNP, with the region sizes centered around this SNP (using GRCh 37 and the biomaRt R package [40,41] to extract genes, gene positions and transcription start sites). Since GTEx only included cis-associations for SNPs +/- 1 Mb of the transcription start site, we only investigated transcripts where the top miscarriage SNP had been analyzed and was within 800 kb of the transcription start site to have sufficient area around each lead SNP to determine colocalization. For the 2 Mb region we used the entire 2 Mb region available in GTEx, whereas we centered the region on the top miscarriage SNP for the smaller region size of 400 kb. The placental genotype data only included summary statistics +/- 100 kb of each gene. Only the miscarriage hit rs143445068 had a gene (*NAV2*) with genotype associations ≥200 kb on each side, which we took forward for colocalization analysis using both all available SNPs as well as a region size of 400 kb centered on the top miscarriage SNP.

We manually inspected LocusCompareR (http://www.locuscompare.com/) plots for all risk locus-gene/protein-tissue combinations where the top available miscarriage SNP was associated with the expression/protein levels at $P<0.05$. One such combination, the rs7859844 risk loci and RP11-154D17.1 expression in visceral adipose tissue, seemed to contain two independent expression quantitative trait loci, including the miscarriage risk loci, albeit not at GWAS-significance threshold. As coloc assumes a maximum of one causal variant per locus [47], we reconducted the colocalization analyses for that analysis using expression statistics conditioned on the top expression variant in GTEx.

We used the prior probabilities ($p1 = 1\times10^{-4}$, $p2 = 1\times10^{-4}$, $p12 = 1\times10^{-6}$) in coloc [47], with the minor allele frequencies from the miscarriage dataset. For INTERVAL and GTEx, we directly supplied effect estimates, standard errors, P-values and the standard deviation in the outcome. There were no effect estimates and standard errors for the miscarriage and placental expression datasets [39]; we supplied P-values for these. We set the threshold for colocalization at a posterior probability for colocalization (PP4) >0.8, for strong colocalization at PP4>0.9, and with a threshold for suggestive evidence of PP4>0.5 [47]. However, no risk locus-gene/protein-tissue combinations had evidence for colocalization.

Supplementary Notes

Supplementary Note 1. Phenotype definition

There is ongoing discussion in the field on how to define different miscarriage phenotypes and distinguish the more severe recurrent miscarriage cases from sporadic miscarriage. According to current (April 2020) ESHRE (European Society on Human Reproduction and Embryology) and ASRM (American Society for Reproductive Medicine) guidelines[48], recurrent miscarriage

(diagnosed with the ICD-10 code N96) is defined as 2 or more spontaneous abortions. However, this has not always been the case, and the most recent set of ESHRE guidelines was published in November 2017. Before that, the recurrent pregnancy loss definition varied from two miscarriages (not necessarily consecutive) [49,50], to three or more consecutive pregnancy losses [51,52]. Of note, the ESHRE 2018 guidelines acknowledged that using two miscarriage as the definition of recurrent miscarriage was at least in part to facilitate research, improve shared decision making with patients and provide them psychological support, rather than because of more specific evidence that this defined a unique phenotype. In fact, the ESHRE guidelines acknowledge not all guideline group members agreed with this definition demonstrating the continuing uncertainty in the field regarding the definition of recurrent miscarriage. Data support increasing numbers of previous miscarriages as being a risk factor for further miscarriage[53] and increasing likelihood of aneuploid miscarriage[54].

All the multiple consecutive miscarriage cohorts (UKBB, EGCUT, ALPSAC) included in this study are from Europe and have received the N96 diagnosis before the updated ESHRE statement in 2017/ 2018 (majority of analyses were conducted in 2017-2018), therefore we combined the N96 diagnosis code with self-reported data for multiple consecutive miscarriage. For all of the reported associated loci (both for sporadic and multiple consecutive miscarriage), we do not see significant heterogeneity across cohorts, i.e. depending on the data used for phenotype definition. Therefore, we believe that at least for the reported associations, the results are not impacted by combining self-reported and electronic health record-derived cases.

To further assess which would be the most appropriate cut-off for defining the sporadic miscarriage phenotype, we used the UKBB data (which is the largest contributing cohort) to evaluate the effect of having a different number of miscarriages on fertility (in this context

measured a) number of pregnancies and b) as the number of children (Supplementary Figure 1). Due to the definitions used to extract cases with 3 or more consecutive miscarriages from self-reported data, this group has less children compared to the other groups and thus represents a miscarriage subphenotype with a more severe effect on fertility, compared to the group with 3 or more miscarriages that are not necessarily consecutive. Similarly, groups with one or two miscarriages are relatively similar in terms of distribution of number of children.

We used the UKBB dataset also to explore the associations between the four identified signals and different miscarriage phenotypes ("1 miscarriage", "2 miscarriages", "3 or more miscarriages", "3 or more consecutive miscarriages (multiple consecutive miscarriage)" (Table S16). rs146350366 identified in sporadic miscarriage analysis was nominally significant in both the "1 miscarriage" ($P= 9.2\times10^{-5}$) and "2 miscarriages" (P=0.017) analyses, but were statistically not significant (and had opposite effect direction) in the 3 or more miscarriage analyses. Two (rs7859844, rs183453668) of the three signals from multiple consecutive miscarriage analyses were nominally significant also in the "3 or more miscarriages" analyses (P=0.01 and P=0.04, respectively), but showed no evidence of association with the "1 miscarriage" and "2 miscarriages" phenotypes. Collectively, this indicates that for the reported associations, at least in the UKBB dataset, the "2 miscarriages" phenotype is more similar to the "1 miscarriage" group than the subset of women who have three or more miscarriages and justifies the joint analysis of women with 1-2 miscarriages.

Supplementary Table 1. Associations between the four signals identified in sporadic miscarriage and multiple consecutive miscarriage meta-analyses and different miscarriage phenotypes in the UKBB

Variant	UKBB			
	1 miscarriage *(n_cases=29414)*	2 miscarriages *(n_cases=7238)*	3 or more miscarriages *(n_cases=3890)*	3 or more consecutive miscarriages *(n_cases=420)*
**Sporadic (1-2) miscarriage meta-analysis**				
**rs146350366**	p=9.2e-05 OR=1.38 (1.18-1.45)	p=0.017 OR=1.45 (1.08-1.94)	p=0.087 OR=0.70 (0.46-1.05)	p=0.051 OR=0.30 (0.09 – 1.005)
**Multiple consecutive (≥ 3) miscarriage meta-analysis**				
**rs7859844**	p=0.27 OR=1.02 (0.98-1.06)	p=0.83 OR=0.99 (0.93-1.06)	p=0.01 OR= 1.13 (1.03-1.24)	p=4.8e-08 OR=2.29 (1.71-3.08)
**rs143445068**	p=0.97 OR=0.99 (0.89-1.11)	p=0.60 OR=1.06 (0.86-1.31)	p=0.06 OR=1.31 (0.98-1.74)	p=3.5e-05 OR=8.3 (3.1-22.6)
**rs183453668**	p=0.13 OR=1.11 (0.97-1.28)	p=0.56 OR=0.92 (0.70-1.21)	p=0.04 OR=1.48 (1.02-2.14)	p=2.9e-04 OR1.9 (3.15-45.28)

Given the above, in this paper we aimed to capture the ends of the miscarriage phenotypic spectrum and contrast sporadic miscarriage (defined here as 1-2 miscarriages) with very severe multiple consecutive miscarriage (3 or more consecutive miscarriages). We have chosen not to use the term “recurrent miscarriage” for the latter group, as it might be confusing for readers in light of the current definition guidelines, which differ from those used in the past.

Supplementary Note 2. Full list of acknowledgements

UKBB

The research in this paper has been carried out using the UK Biobank resource (under applications 17805 (“Dissemination of shared genetics across phenotypes associated with

reproductive health and related endophenotypes”), 11867 (“Dissection of the Genetic Susceptibility of Obesity Traits and their Comorbidities”), and 16729 (“MR-PheWAS: hypothesis prioritization among potential causal effects of body mass index on many outcomes, using Mendelian randomization”)).

EGCUT

This study was funded by EU H2020 grant 692145, Estonian Research Council Grant IUT20-60, IUT24-6, and European Union through the European Regional Development Fund Project No. 2014-2020.4.01.15-0012 GENTRANSMED and 2014-2020.4.01.16-0125. Data analyses were carried out in part in the High-Performance Computing Center of University of Tartu.

iPSYCH

iPSYCH2012 has been conducted using the Danish National Biobank resource, supported by the Novo Nordisk Foundation.

QIMR

We thank the twins and their families for their participation in the QIMR study. Funding was provided by the Australian National Health and Medical Research Council (241944, 339462, 389927, 389875, 389891, 389892, 389938, 442915, 442981, 496739, 552485, 552498, 1084325), the Australian Research Council (A7960034, A79906588, A79801419, DP0770096, DP0212016, DP0343921), the FP-5 GenomEUtwin Project (QLG2-CT-2002-01254), and the U.S. National Institutes of Health (NIH grants AA07535, AA10248, AA13320, AA13321,

AA13326, AA14041, MH66206). S.E.M. is supported by a National Health and Medical Research Council (NHMRC) Senior Research Fellowship (1103623).

QIMREndo

We acknowledge with appreciation all women who participated in the QIMR endometriosis study. This study was supported by grants from the Australian National Health and Medical Research Council (241944, 339462, 389927,389875, 389891, 389892, 389938, 443036, 442915, 442981, 496610, 496739, 552485, 552498), the Cooperative Research Centre for Discovery of Genes for Common Human Diseases (CRC), Cerylid Biosciences (Melbourne), and donations from Neville and Shirley Hawkins. D.R.N and G.W.M are supported by the NHMRC Fellowship Scheme.

ALSPAC

We are extremely grateful to all the families who took part in the ALSPAC study, the midwives for their help in recruiting them, and the whole ALSPAC team, which includes interviewers, computer and laboratory technicians, clerical workers, research scientists, volunteers, managers, receptionists and nurses. FUNDING: This study was supported by the US National Institute of Health (R01 DK10324), which also supports A.L.G.S' salary, the European Research Council under the European Union's Seventh Framework Programme (FP7/2007-2013) / ERC grant agreement no 669545 and the NIHR Biomedical Centre at the University Hospitals Bristol NHS Foundation Trust and the University of Bristol. Core funding for ALSPAC is provided by the UK Medical Research Council and Wellcome (Grant ref: 102215/2/13/2) and the University of Bristol. Genotyping of the ALSPAC maternal samples was

funded by the Wellcome Trust (WT088806). A comprehensive list of grants funding is available on the ALSPAC website (http://www.bristol.ac.uk/alspac/external/documents/grant-acknowledgements.pdf). A.L.G.S and D.A.L work in a Unit that receives support from the University of Bristol and UK Medical Research Council (MC_UU_00011/6). None of the funders influenced the analyses or interpretation of results. The views expressed in this paper are those of the authors and not necessarily any funding body. D.A.L and A.L.G.S will serve as guarantors for the results from ALSPAC.

WHI

The WHI program is funded by the National Heart, Lung, and Blood Institute, National Institutes of Health, U.S. Department of Health and Human Services through contracts HHSN268201600018C, HHSN268201600001C, HHSN268201600002C, HHSN268201600003C, and HHSN268201600004C. This manuscript was not prepared in collaboration with investigators of the WHI, has not been reviewed and/or approved by the Women's Health Initiative (WHI), and does not necessarily reflect the opinions of the WHI investigators or the NHLBI. Funding support for WHI GARNET was provided through the NHGRI Genomics and Randomized Trials Network (GARNET) (Grant Number U01 HG005152). Assistance with phenotype harmonization and genotype cleaning, as well as with general study coordination, was provided by the GARNET Coordinating Center (U01 HG005157). Assistance with data cleaning was provided by the National Center for Biotechnology Information. Funding support for genotyping, which was performed at the Broad Institute of MIT and Harvard, was provided by the NIH Genes, Environment and Health Initiative [GEI] (U01 HG004424). Funding for WHI SHARe genotyping was provided by

NHLBI Contract N02-HL-64278. The WHI datasets used for the analyses described in this manuscript were obtained from dbGaP at http://www.ncbi.nlm.nih.gov/sites/entrez?db=gap through dbGaP accessions phs000200.v11.p3.c1 and phs000200.v11.p3.c2.

Partners HealthCare Biobank

Partners HealthCare Biobank is supported by NHGRI grant U01HG008685.

Lifelines

The Lifelines Cohort Study, and generation and management of GWAS genotype data for the Lifelines Cohort Study is supported by the Netherlands Organization of Scientific Research NWO (grant 175.010.2007.006), the Economic Structure Enhancing Fund (FES) of the Dutch government, the Ministry of Economic Affairs, the Ministry of Education, Culture and Science, the Ministry for Health, Welfare and Sports, the Northern Netherlands Collaboration of Provinces (SNN), REP (Ruimtelijk Economisch Programma), the Province of Groningen, University Medical Center Groningen, the University of Groningen, Dutch Kidney Foundation and Dutch Diabetes Research Foundation.

MoBa

This work was supported by grants from the European Research Council (AdG #293574), the Bergen Research Foundation ("Utilizing the Mother and Child Cohort and the Medical Birth Registry for Better Health"), Stiftelsen Kristian Gerhard Jebsen (Translational Medical Center), the University of Bergen, the Research Council of Norway (FRIPRO grant #240413), the Western Norway Regional Health Authority (Strategic Fund "Personalized Medicine for

Children and Adults”), and the Norwegian Diabetes Foundation; and Helse Vest's Open Research Grant. This work was partly supported by the Research Council of Norway through its Centres of Excellence funding scheme (#262700), Better Health by Harvesting Biobanks (#229624) and The Swedish Research Council, Stockholm, Sweden (2015-02559), The Research Council of Norway, Oslo, Norway (FRIMEDBIO ES547711, March of Dimes (#21-FY16-121). The Norwegian Mother and Child Cohort Study is supported by the Norwegian Ministry of Health and Care Services and the Ministry of Education and Research, NIH/NIEHS (contract no N01-ES-75558), NIH/NINDS (grant no.1 UO1 NS 047537-01 and grant no.2 UO1 NS 047537-06A1). We are grateful to all the families in Norway who are taking part in this ongoing cohort study.

Kadoorie Biobank

The CKB baseline survey and the first re-survey were supported by the Kadoorie Charitable Foundation in Hong Kong. Long-term follow-up was supported by the UK Wellcome Trust (202922/Z/16/Z, 104085/Z/14/Z, 088158/Z/09/Z), the National Natural Science Foundation of China (81390540, 81390541, 81390544), and the National Key Research and Development Program of China (2016YFC0900500, 2016YFC0900501, 2016YFC0900504, 2016YFC1303904). DNA extraction and genotyping were supported by GlaxoSmithKline and the UK Medical Research Council (MC-PC-13049, MC-PC-14135). The project was supported by British Heart Foundation, UK Medical Research Council and Cancer Research provide core funding to the Clinical Trial Service Unit and Epidemiological Studies Unit at Oxford University.

Supplementary Figures

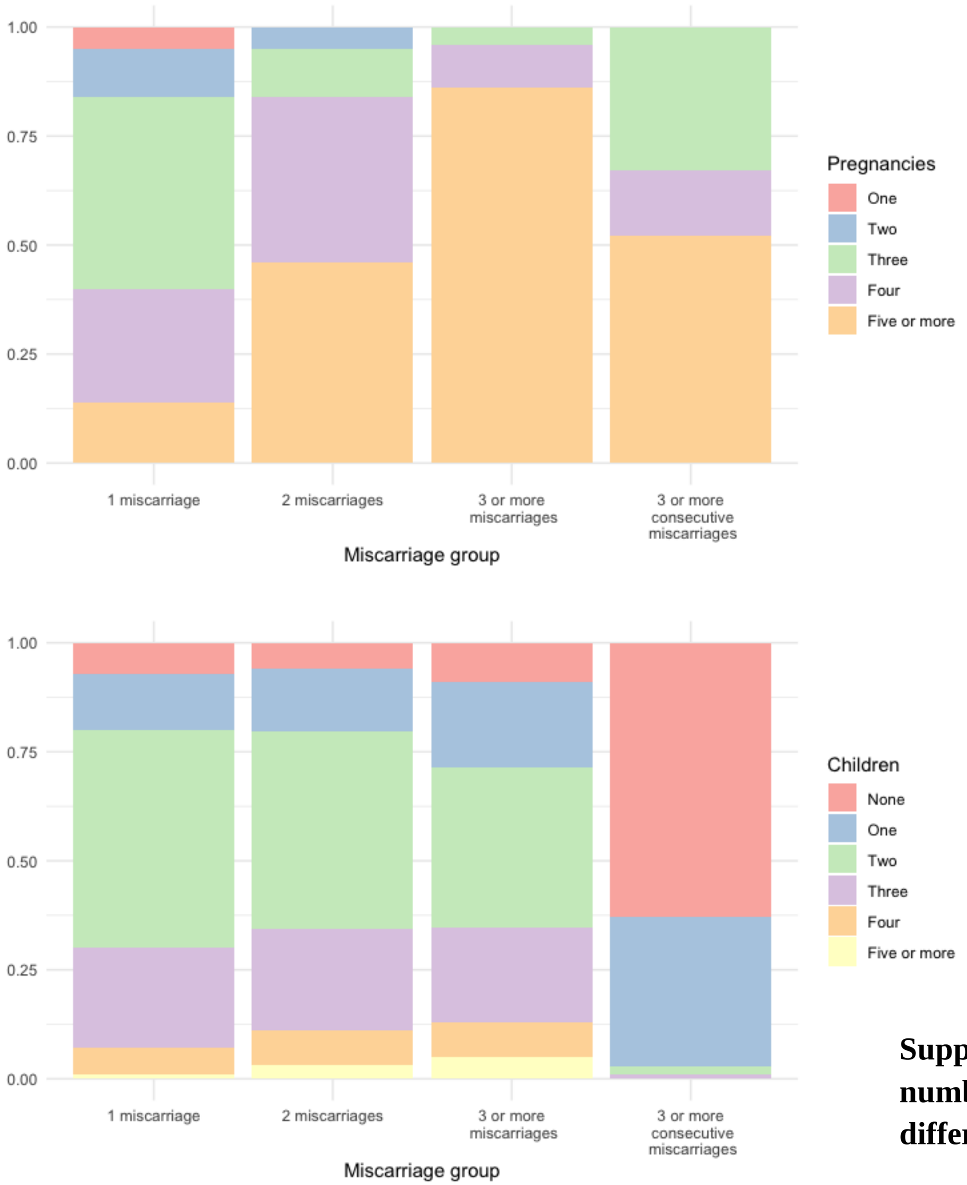

Supplementary Figure 1. Proportional distribution of a) number of pregnancies and b) number of children in different miscarriage subphenotypes.

A

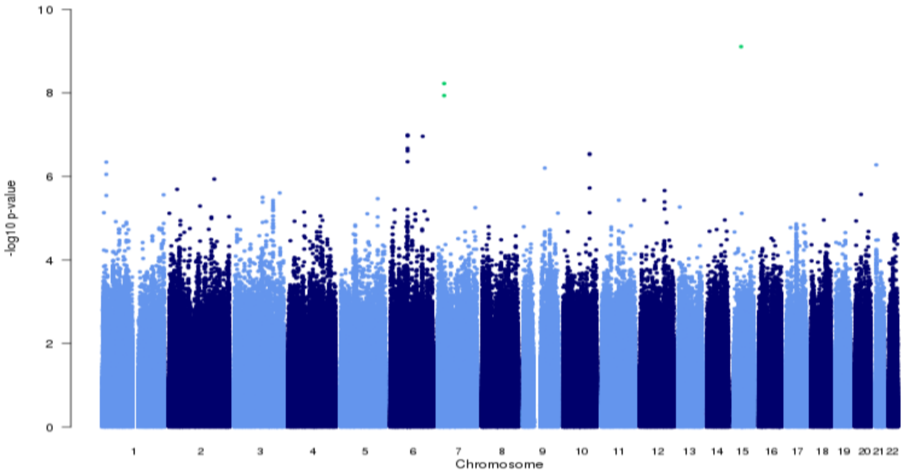

B

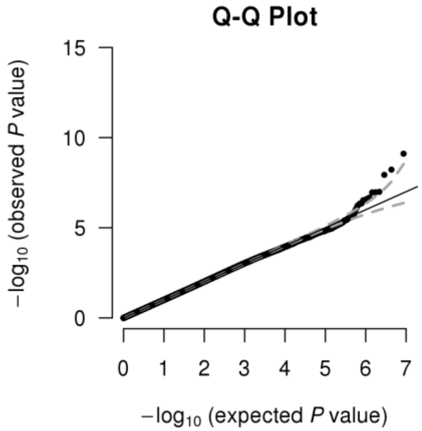

C

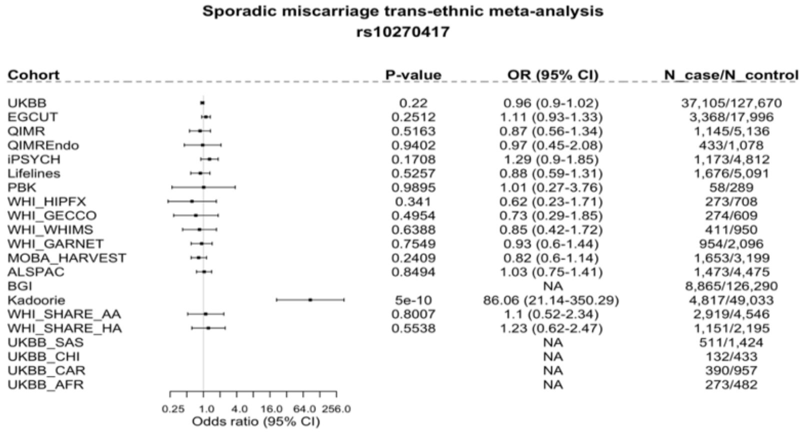

Supplementary Figure 2. Manhattan plot (A) and QQ plot with 95% confidence interval (B) for sporadic miscarriage trans-ethnic meta-analysis (69,054 cases and 359,469) for markers present in at least half (n=11) of the cohorts. Genome-wide significance threshold was set at p-value = 5×10^{-8}.

C Forest plot of the association signal on chromosome 7 (rs10270417). Details of association testing in individual cohorts can be found in Supplementary Data 2. OR estimates with 95% confidence intervals (error bars) have been plotted.

A

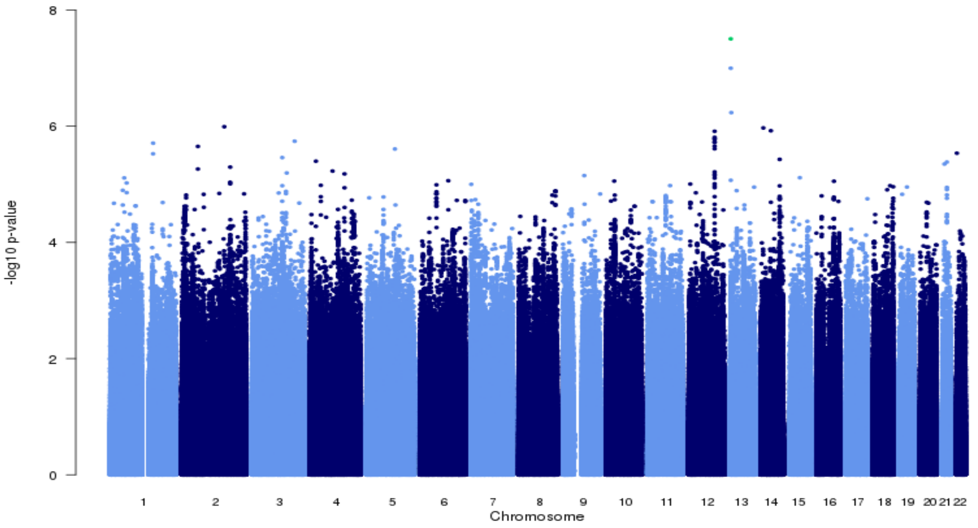

B

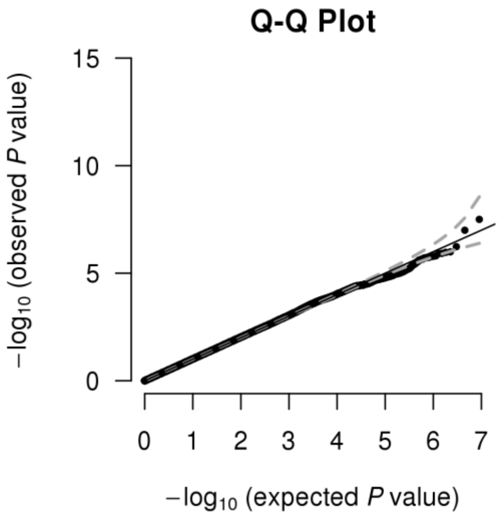

C

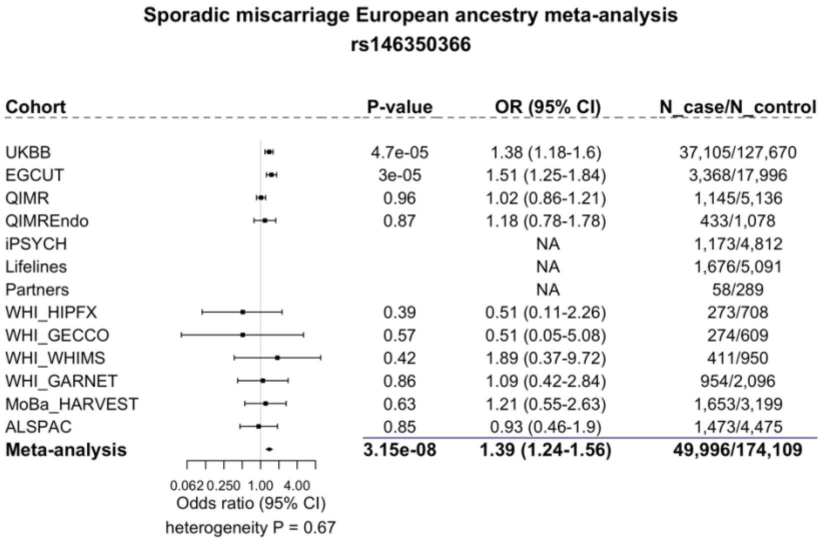

Supplementary Figure 3. Manhattan (A), and QQ plot with 95% confidence interval (B) for sporadic miscarriage European ancestry meta-analysis (49,996 cases and 174,109 controls) filtered for markers present in at least half (n=7) of the cohorts. The genome-wide significant marker on chromosome 13 (rs146350366) is highlighted in green on the Manhattan plot. Genome-wide significance threshold was set at p-value = 5×10^{-8}. **C Forest plot of the association signal on chromosome 13 (rs146350366).** Details of association testing in individual cohorts can be found in Supplementary Data 2. Summary effect estimate from inverse variance fixed effects meta-analysis. OR estimates with 95% confidence intervals (error bars) have been plotted.

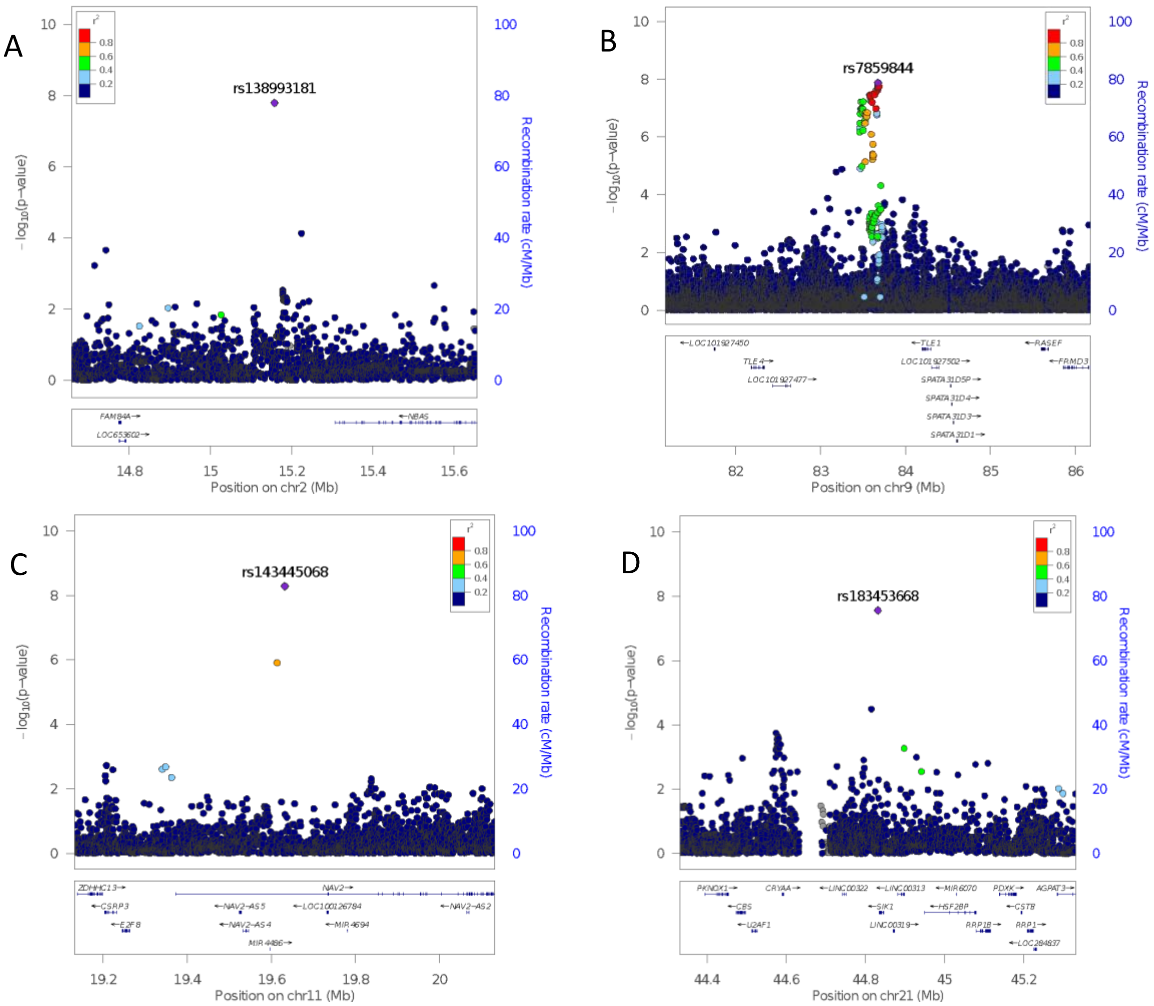

Supplementary Figure 4. Regional plots for multiple consecutive miscarriage European ancestry meta-analysis top hits. A) rs138993181 on chromosome 2; B) rs7859844 on chromosome 9; C) rs143445068 on chromosome 11; D) rs183453668 on chromosome 21.

A

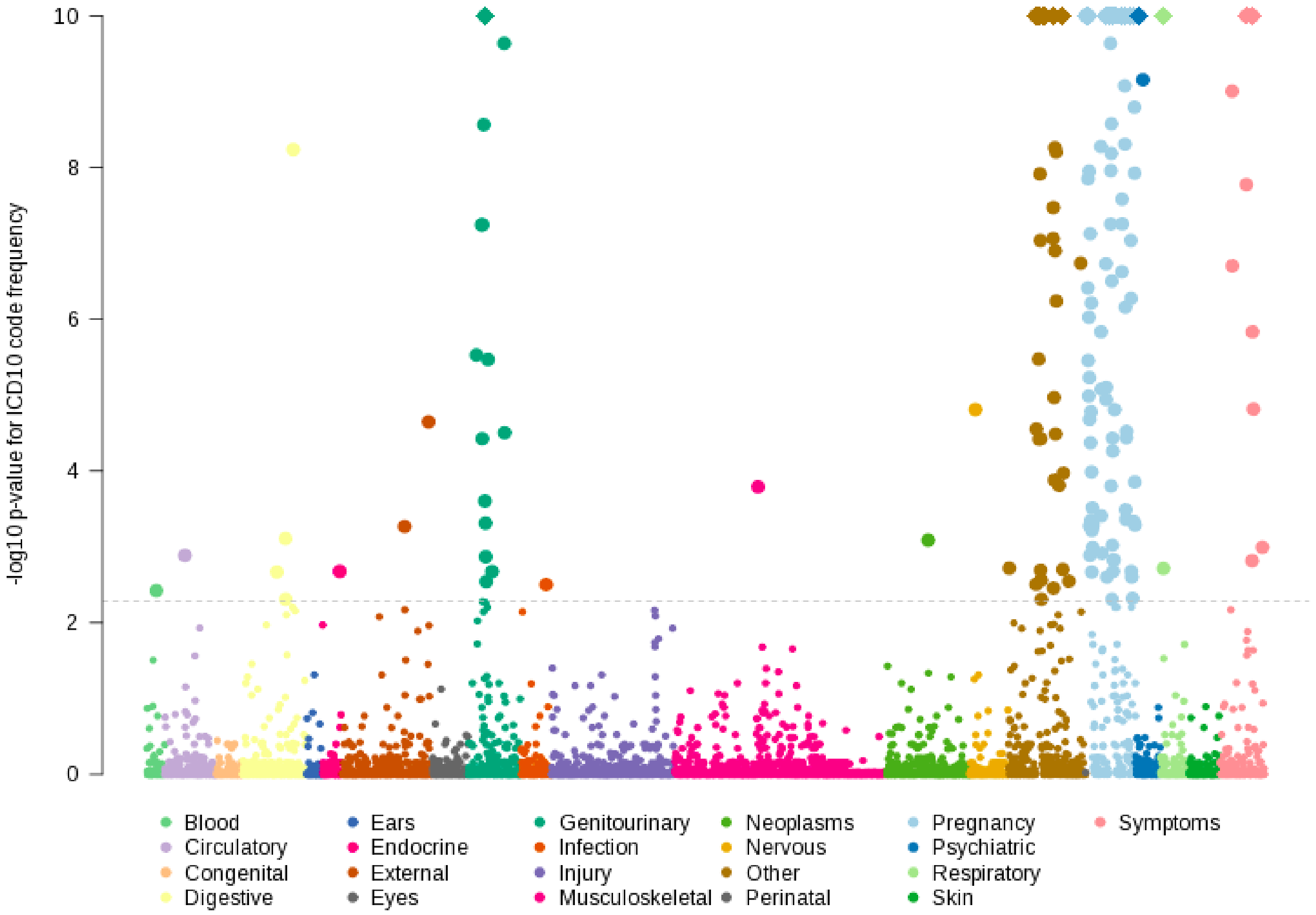

-log10 p-value for ICD10 code frequency
0
2
4
6
8
10
Blood
Circulatory
Congenital
Digestive
Ears
Endocrine
External
Eyes
Genitourinary
Infection
Injury
Musculoskeletal
Neoplasms
Nervous
Other
Perinatal
Pregnancy
Psychiatric
Respiratory
Skin
Symptoms

B

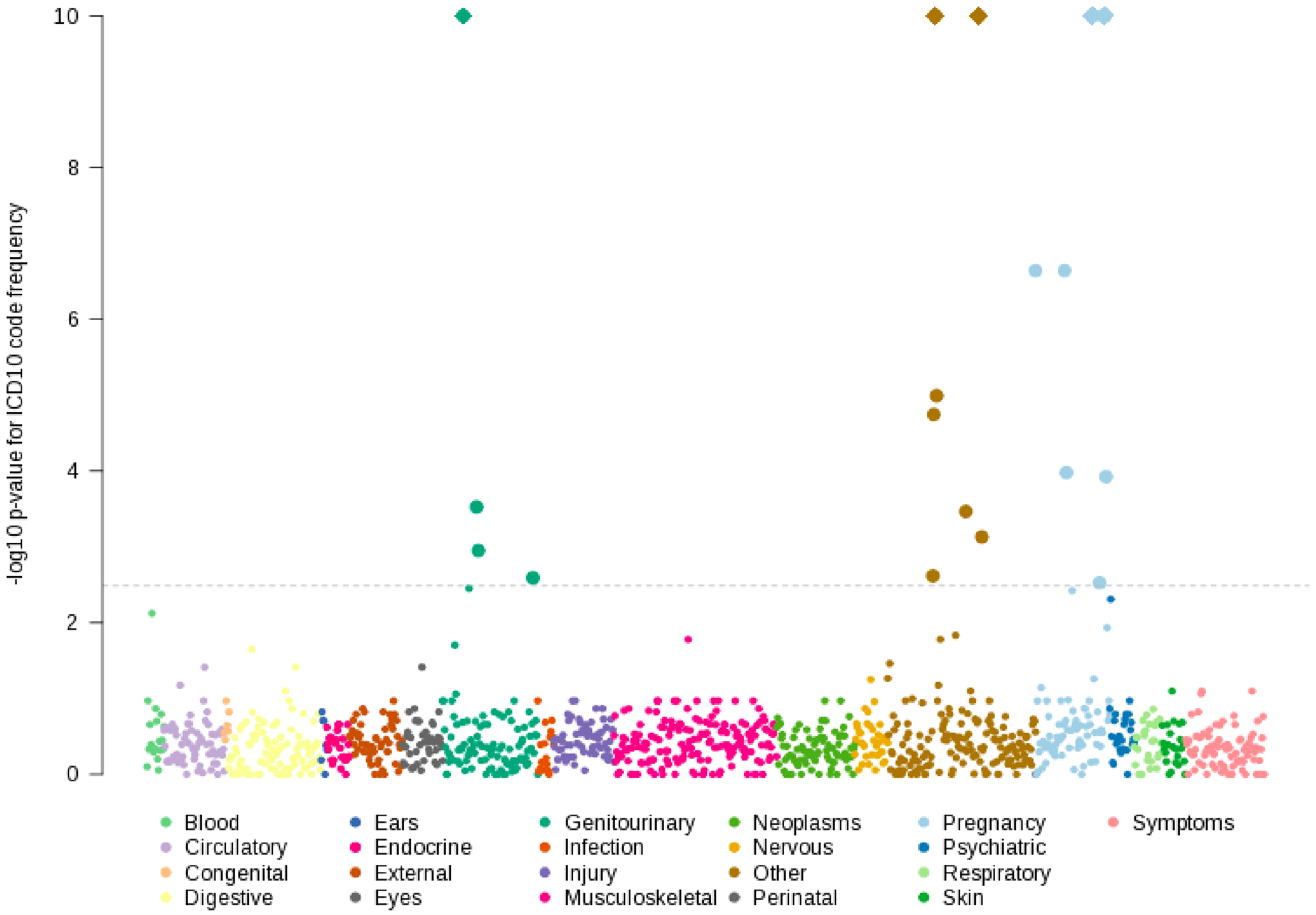

Supplementary Figure 5. ICD codes associated with sporadic (A) and multiple consecutive (B) miscarriage (unadjusted analysis). Each point in the plot represents one

ICD10 code observed among the cases. Y-axis is the –log10 *P*-value of the test of difference between the diagnosis frequency among cases and controls. Diagnoses were grouped and coloured by the ICD10 chapter. Y-axis is truncated at 10, with diagnosis for which its –log10 *P*-value exceeds this value represented by a diamond. Horizontal dash line represents the significance threshold after applying the 10% FDR multiple testing correction (5.23×10^{-3} for sporadic and 3.28×10^{-3} for multiple consecutive miscarriage, respectively).

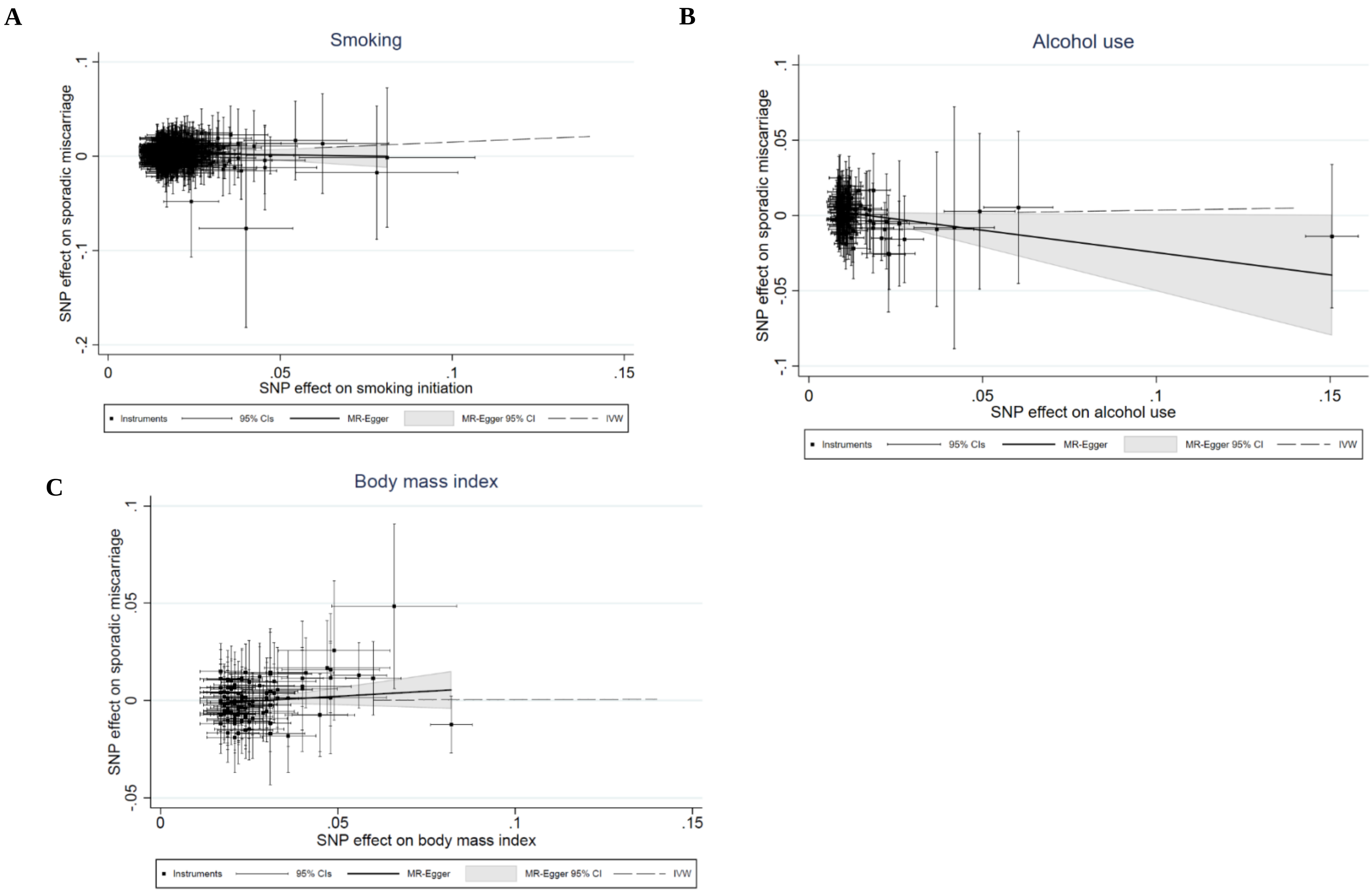

Supplementary Figure 6. Two sample Mendelian randomization analysis. Instruments-sporadic miscarriage associations (y-axis) against instrument-smoking (a), instrument-alcohol (b), and instrument-body mass index (c) associations (x-axis). Error bars denote 95% confidence intervals for effect estimates.

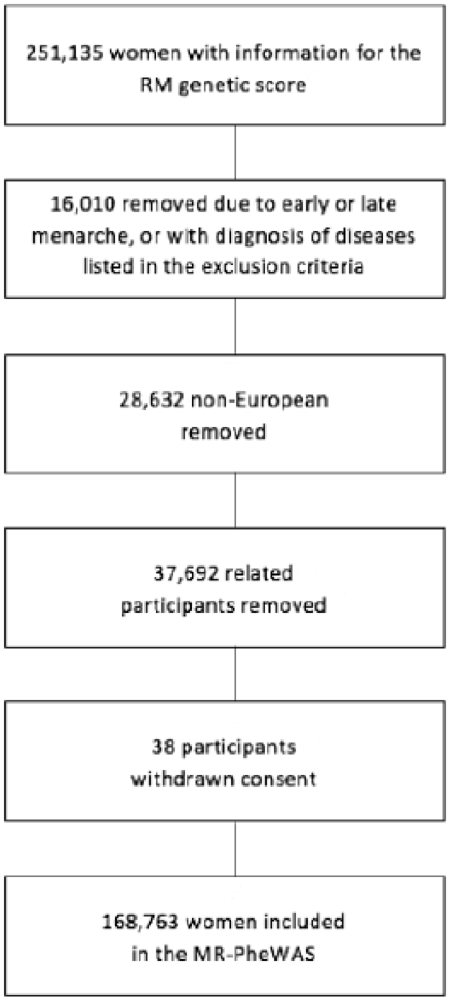

Supplementary Figure 7. Participant flow diagram for the MR-PheWAS

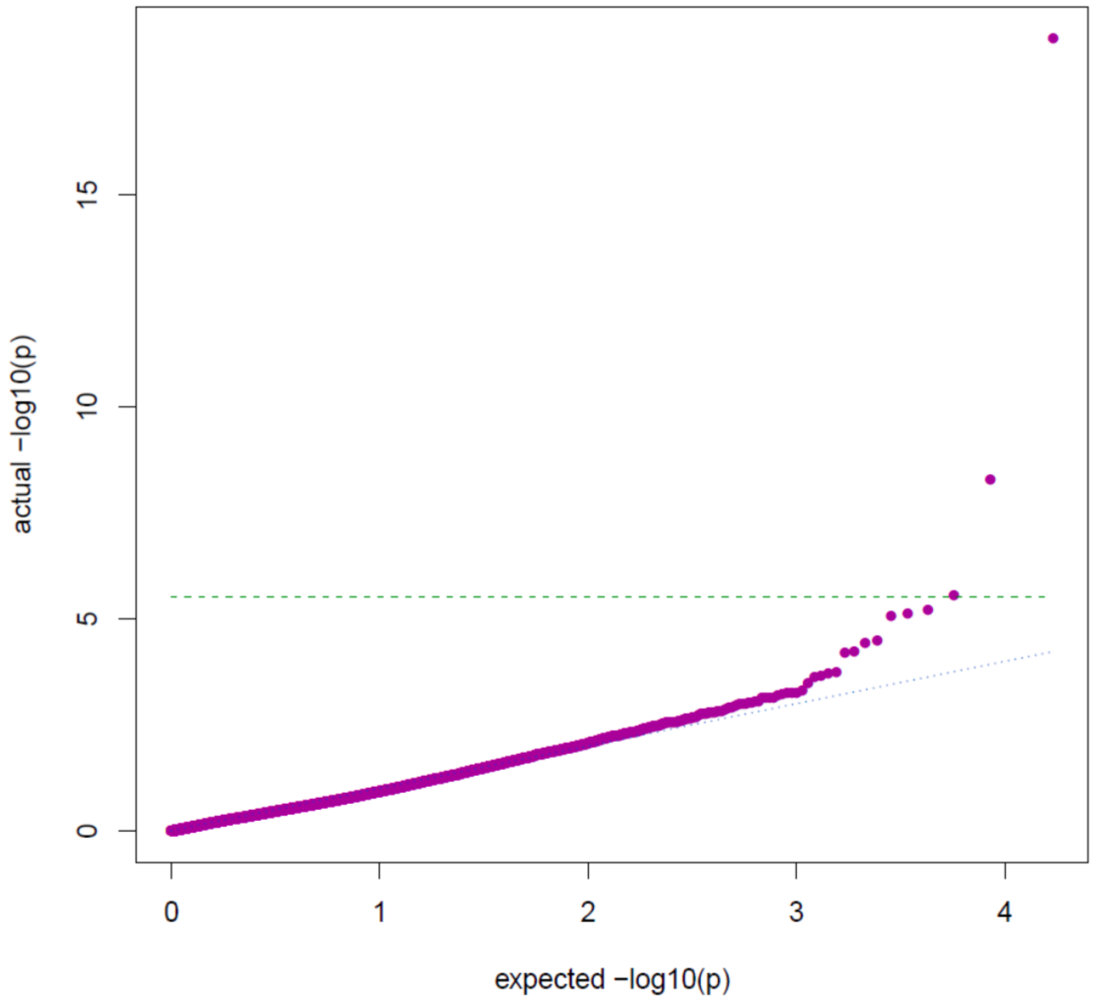

Supplementary Figure 8. QQ plot for the 17,028 MR-PheWAS results for multiple consecutive miscarriage genetic risk score. The three significant outcomes are single items from multi-item categories, with none of the other items reaching suggestive thresholds of statistical significance.

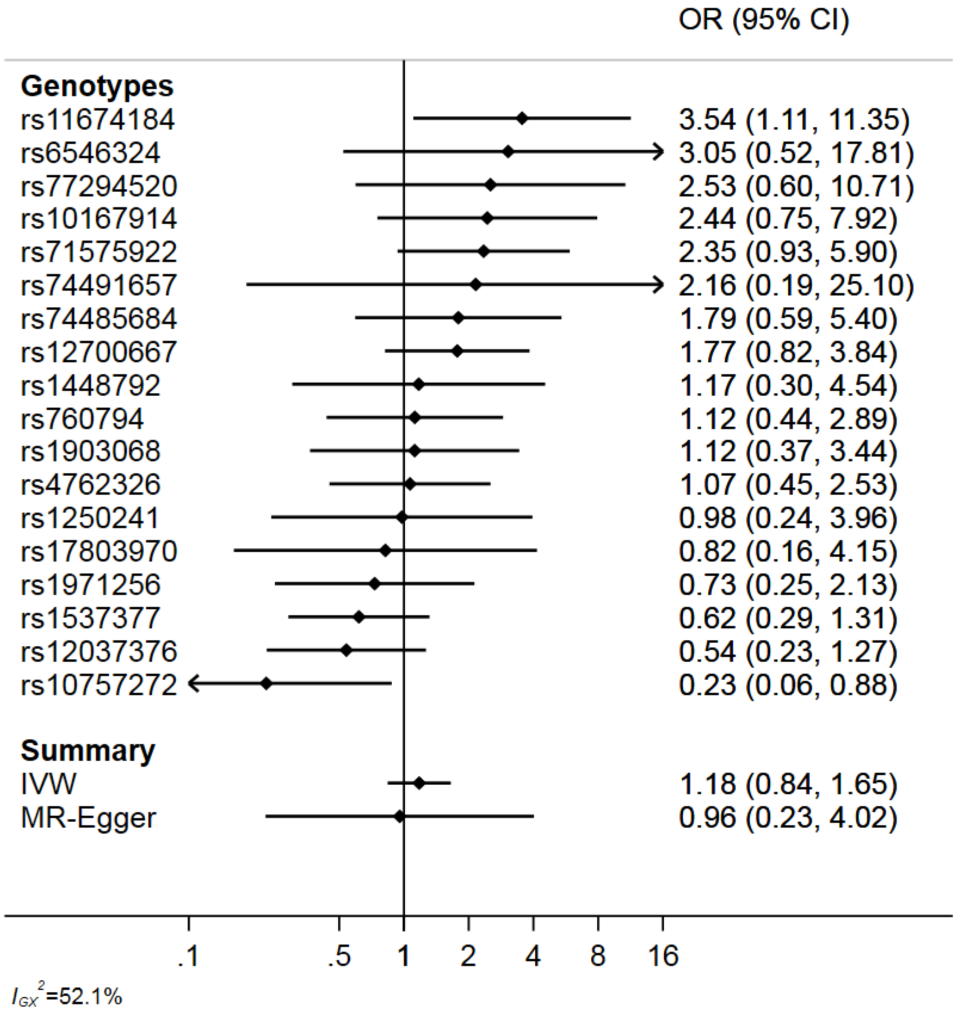

Supplementary Figure 9. Forest plot showing endometriosis risk allele effects for multiple consecutive miscarriage and pooled effect according to inverse variance weighting and MR-Egger methods. Error bars denote 95% confidence intervals for effect estimate.

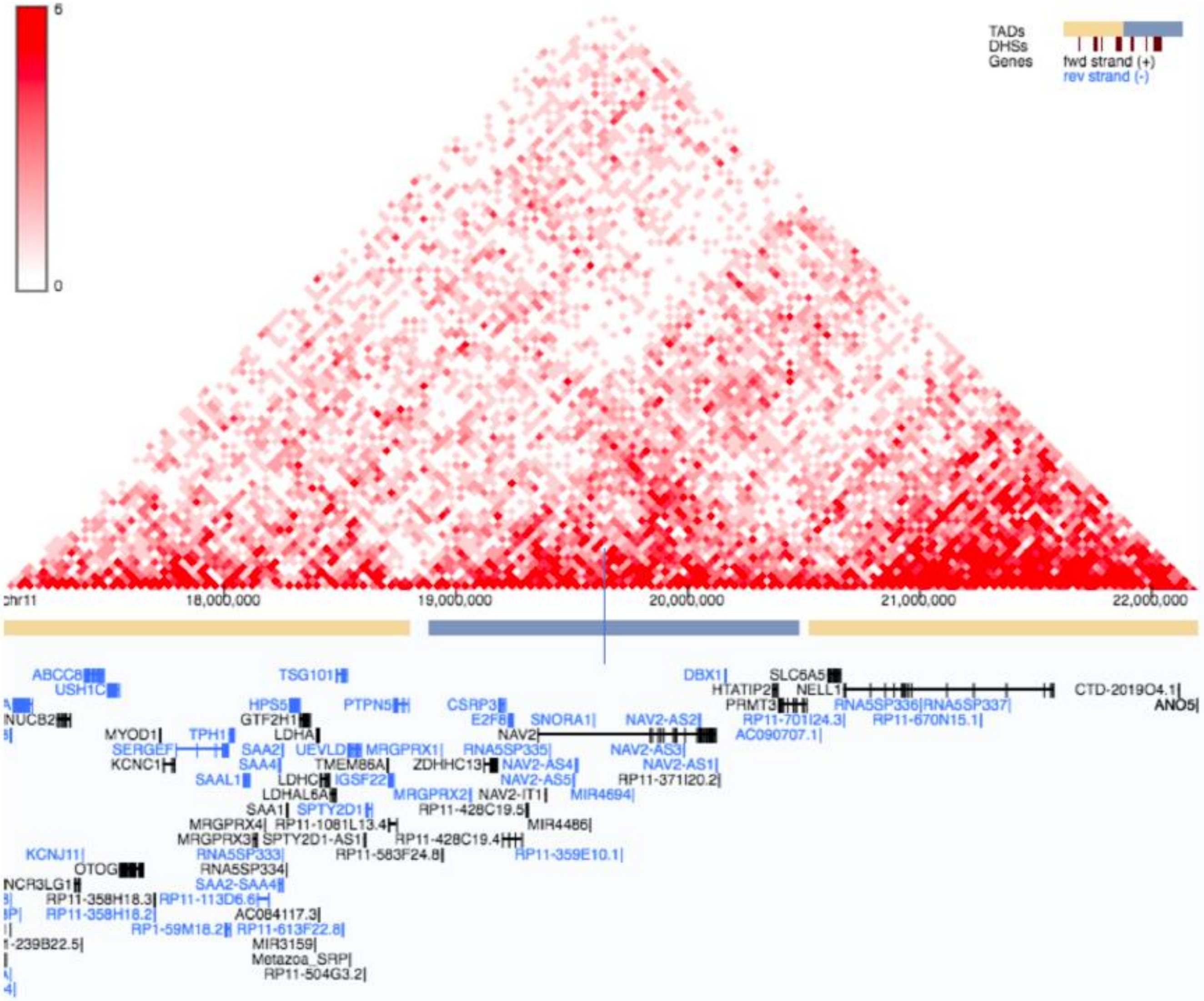

5
0
TADs
DHSs
Genes
fwd strand (+)
rev strand (-)
chr11
18,000,000
19,000,000
20,000,000
21,000,000
22,000,000
ABCC8
USH1C
TSG101
DBX1
SLC6A5
HTATIP2
NELL1
CTD-2019O4.1
ANO5
HPS5
PTPN5
CSRP3
PRMT3
RNA5SP336
RNA5SP337
NUCB2
GTF2H1
E2F8
SNORA1
NAV2-AS2
RP11-701I24.3
RP11-670N15.1
MYOD1
TPH1
LDHA
NAV2
AC090707.1
SERGEF
SAA2
UEVLD
MRGPRX1
RNA5SP335
NAV2-AS3
KCNC1
SAA4
TMEM86A
ZDHHC13
NAV2-AS4
NAV2-AS1
SAAL1
LDHC
IGSF22
NAV2-AS5
RP11-371I20.2
LDHAL6A
MRGPRX2
NAV2-IT1
MIR4694
SAA1
SPTY2D1
RP11-428C19.5
MRGPRX4
RP11-1081L13.4
MIR4486
MRGPRX3
SPTY2D1-AS1
RP11-428C19.4
KCNJ11
RNA5SP333
RP11-583F24.8
RP11-359E10.1
OTOG
RNA5SP334
NCR3LG1
SAA2-SAA4
RP11-358H18.3
RP11-113D6.6
RP11-358H18.2
AC084117.3
RP1-59M18.2
RP11-613F22.8
MIR3159
Metazoa_SRP
RP11-504G3.2

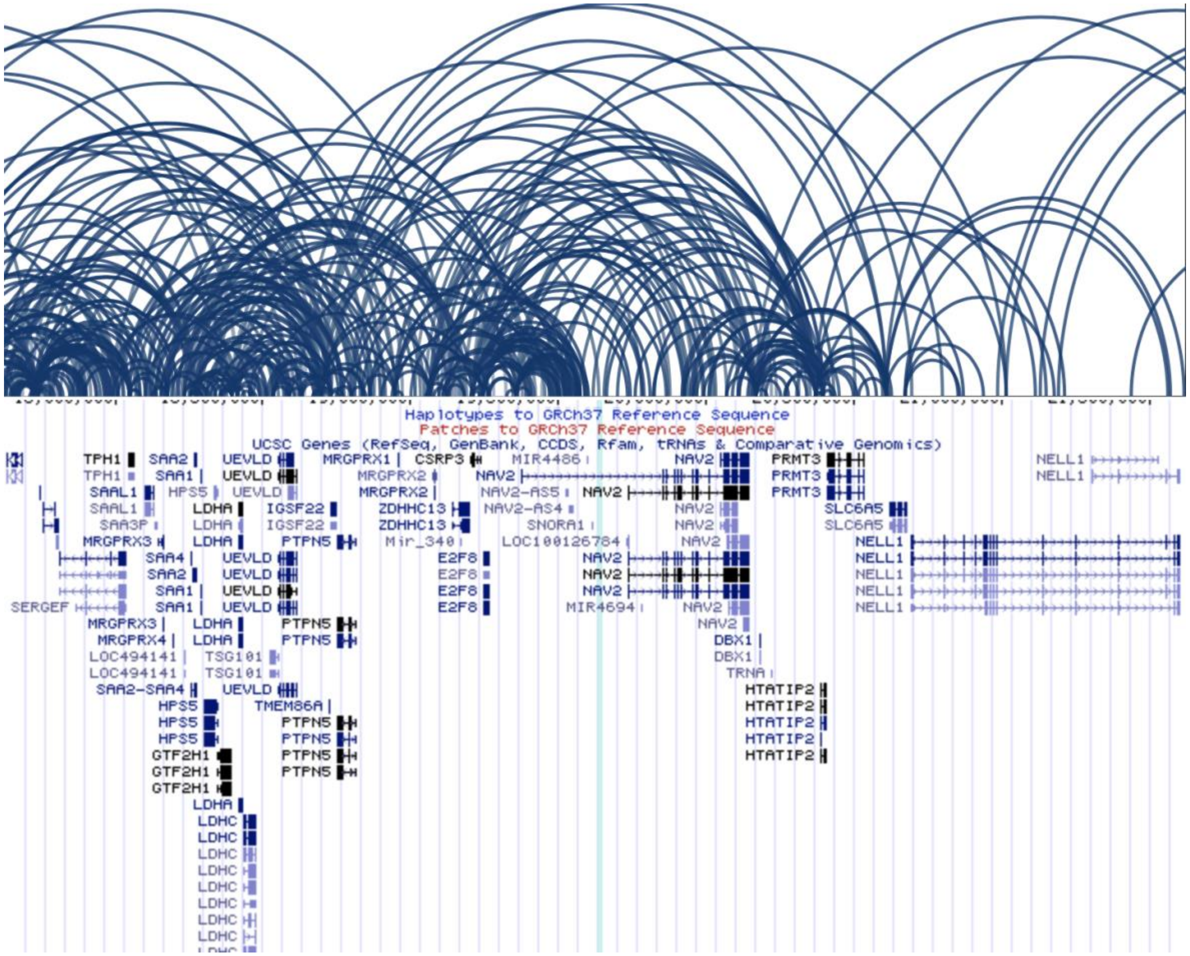

Supplementary Figure 10. Hi-C map and Capture Hi-C data visualization for the multiple consecutive miscarriage association signal on chromosome 11. The blue vertical line represents the location of the signal from GWAS meta-analysis. The 3D Genome Browser[31] was used for data visualization and ovarian tissue and trophoblast cell data were selected to illustrate the chromatin architecture at the locus, respectively.

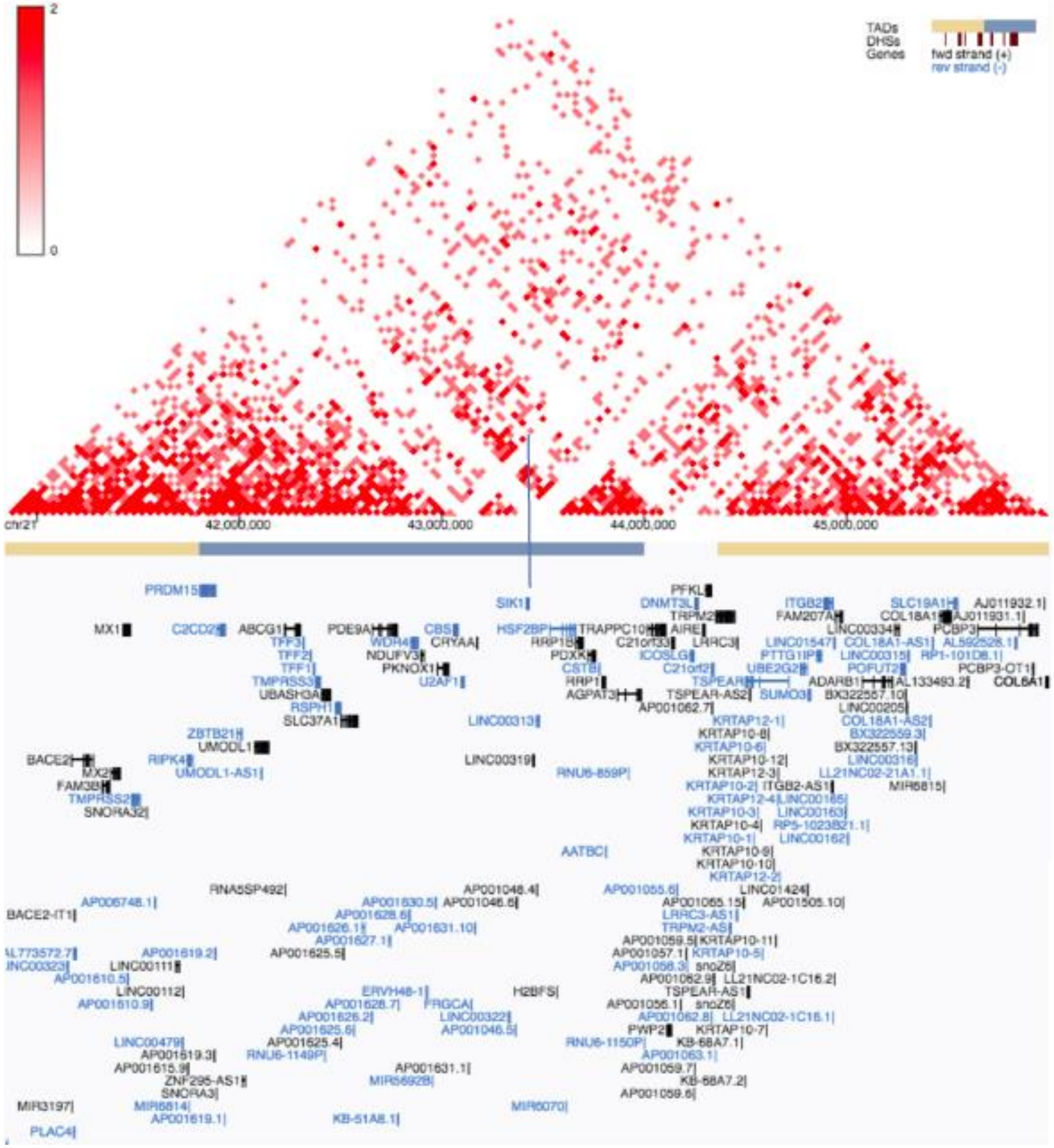
2
0
TADs
DHSs
Genes
fwd strand (+)
rev strand (-)
chr21
42,000,000
43,000,000
44,000,000
45,000,000

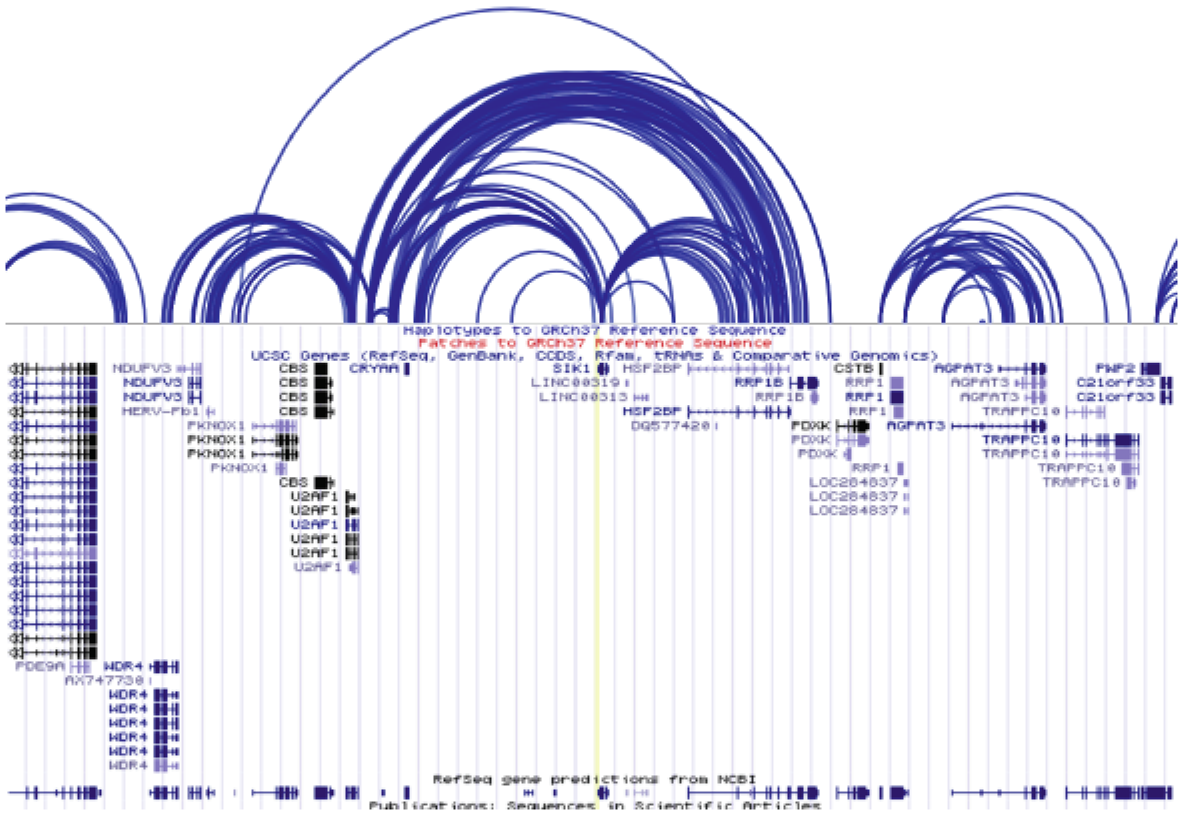

Supplementary Figure 11. Hi-C map and Capture Hi-C data visualization for multiple consecutive miscarriage association signal on chr21. The blue/yellow vertical line represents the location of the signal from GWAS meta-analysis. The 3D Genome Browser[31] was used for data visualization and ovarian tissue and endothelial progenitors were selected to illustrate the Hi-C data and Capture Hi-C data at the locus, respectively.

Supplementary References

1. Sudlow, C. *et al.* UK Biobank: An Open Access Resource for Identifying the Causes of a Wide Range of Complex Diseases of Middle and Old Age. *PLoS Med.* **12**, e1001779 (2015).
2. Leitsalu, L. *et al.* Cohort Profile: Estonian Biobank of the Estonian Genome Center, University of Tartu. *Int. J. Epidemiol.* **44**, 1137–1147 (2015).
3. Fraser, A. *et al.* Cohort Profile: the Avon Longitudinal Study of Parents and Children: ALSPAC mothers cohort. *Int. J. Epidemiol.* **42**, 97–110 (2013).
4. Medland, S. E. *et al.* Males Do Not Reduce the Fitness of Their Female Co-Twins in Contemporary Samples. *Twin Res. Hum. Genet.* **11**, 481–487 (2008).
5. Painter, J. N. *et al.* Genome-wide association study identifies a locus at 7p15.2 associated with endometriosis. *Nat. Genet.* **43**, 51–54 (2011).
6. Treloar, S. A. *et al.* Genomewide linkage study in 1,176 affected sister pair families identifies a significant susceptibility locus for endometriosis on chromosome 10q26. *Am. J. Hum. Genet.* **77**, 365–76 (2005).
7. Pedersen, C. B. *et al.* The iPSYCH2012 case-cohort sample: New directions for unravelling genetic and environmental architectures of severe mental disorders. *Molecular Psychiatry* **23**, 6–14 (2018).
8. Stolk, R. P. *et al.* Universal risk factors for multifactorial diseases: LifeLines: a three-generation population-based study. *Eur. J. Epidemiol.* **23**, 67–74 (2008).
9. Scholtens, S. *et al.* Cohort Profile: LifeLines, a three-generation cohort study and biobank. *Int. J. Epidemiol.* **44**, 1172–80 (2015).
10. Rønningen, K. S. *et al.* The biobank of the Norwegian mother and child cohort Study: A resource for the next 100 years. *Eur. J. Epidemiol.* **21**, 619–625 (2006).

11. Magnus, P. *et al.* Cohort Profile Update: The Norwegian Mother and Child Cohort Study (MoBa). *Int. J. Epidemiol.* **45**, 382–388 (2016).

12. Magnus, P. *et al.* Cohort profile: The Norwegian Mother and Child Cohort Study (MoBa). *Int. J. Epidemiol.* **35**, 1146–1150 (2006).

13. Liu, S. *et al.* Genomic Analyses from Non-invasive Prenatal Testing Reveal Genetic Associations, Patterns of Viral Infections, and Chinese Population History. *Cell* **175**, 347-359.e14 (2018).

14. Chen, Z. *et al.* China Kadoorie Biobank of 0.5 million people: survey methods, baseline characteristics and long-term follow-up. *Int. J. Epidemiol.* **40**, 1652–66 (2011).

15. de Leeuw, C. A., Mooij, J. M., Heskes, T. & Posthuma, D. MAGMA: generalized gene-set analysis of GWAS data. *PLoS Comput. Biol.* **11**, e1004219 (2015).

16. Watanabe, K., Taskesen, E., van Bochoven, A. & Posthuma, D. Functional mapping and annotation of genetic associations with FUMA. *Nat. Commun.* **8**, 1826 (2017).

17. Watanabe, K. *et al.* A global view of pleiotropy and genetic architecture in complex traits. *bioRxiv* 500090 (2018). doi:10.1101/500090

18. Roederer, M. *et al.* The genetic architecture of the human immune system: a bioresource for autoimmunity and disease pathogenesis. *Cell* **161**, 387–403 (2015).

19. Wang, K., Li, M. & Hakonarson, H. ANNOVAR: functional annotation of genetic variants from high-throughput sequencing data. *Nucleic Acids Res.* **38**, e164 (2010).

20. Kircher, M. *et al.* A general framework for estimating the relative pathogenicity of human genetic variants. *Nat. Genet.* **46**, 310–5 (2014).

21. Boyle, A. P. *et al.* Annotation of functional variation in personal genomes using RegulomeDB. *Genome Res.* **22**, 1790–7 (2012).

22. Dunham, I. *et al.* An integrated encyclopedia of DNA elements in the human genome.

Nature **489**, 57–74 (2012).

23. Roadmap Epigenomics Consortium, A. *et al.* Integrative analysis of 111 reference human epigenomes. *Nature* **518**, 317–30 (2015).
24. Lonsdale, J. *et al.* The Genotype-Tissue Expression (GTEx) project. *Nat. Genet.* **45**, 580–585 (2013).
25. Westra, H.-J. *et al.* Systematic identification of trans eQTLs as putative drivers of known disease associations. *Nat. Genet.* **45**, 1238–1243 (2013).
26. Zhernakova, D. V *et al.* Identification of context-dependent expression quantitative trait loci in whole blood. *Nat. Genet.* **49**, 139–145 (2016).
27. Ramasamy, A. *et al.* Genetic variability in the regulation of gene expression in ten regions of the human brain. *Nat. Neurosci.* **17**, 1418–1428 (2014).
28. Grundberg, E. *et al.* Mapping cis- and trans-regulatory effects across multiple tissues in twins. *Nat. Genet.* **44**, 1084–1089 (2012).
29. Ng, B. *et al.* An xQTL map integrates the genetic architecture of the human brain's transcriptome and epigenome. *Nat. Neurosci.* **20**, 1418–1426 (2017).
30. Fromer, M. *et al.* Gene expression elucidates functional impact of polygenic risk for schizophrenia. *Nat. Neurosci.* **19**, 1442–1453 (2016).
31. Schmitt, A. *et al.* A Compendium of Chromatin Contact Maps Reveals Spatially Active Regions in the Human Genome. *Cell Rep.* **17**, 2042–2059 (2016).
32. Pierce, B. L. & Burgess, S. Efficient design for Mendelian randomization studies: subsample and 2-sample instrumental variable estimators. *Am. J. Epidemiol.* **178**, 1177–84 (2013).
33. Sapkota, Y. *et al.* Meta-analysis identifies five novel loci associated with endometriosis highlighting key genes involved in hormone metabolism. *Nat. Commun.* **8**, 15539 (2017).

34. Willer, C. J., Li, Y. & Abecasis, G. R. METAL: fast and efficient meta-analysis of genomewide association scans. *Bioinformatics* **26**, 2190–2191 (2010).

35. Burgess, S., Butterworth, A. & Thompson, S. G. Mendelian randomization analysis with multiple genetic variants using summarized data. *Genet. Epidemiol.* **37**, 658–65 (2013).

36. Bowden, J., Davey Smith, G. & Burgess, S. Mendelian randomization with invalid instruments: effect estimation and bias detection through Egger regression. *Int. J. Epidemiol.* **44**, 512–25 (2015).

37. Sun, B. B. *et al.* Genomic atlas of the human plasma proteome. *Nature* **558**, 73–79 (2018).

38. GTEx Consortium *et al.* Genetic effects on gene expression across human tissues. *Nature* **550**, 204–213 (2017).

39. Kikas, T., Rull, K., Beaumont, R. N., Freathy, R. M. & Laan, M. The Effect of Genetic Variation on the Placental Transcriptome in Humans. *Front. Genet.* **10**, 550 (2019).

40. Durinck, S. *et al.* BioMart and Bioconductor: a powerful link between biological databases and microarray data analysis. *Bioinformatics* **21**, 3439–40 (2005).

41. Durinck, S., Spellman, P. T., Birney, E. & Huber, W. Mapping identifiers for the integration of genomic datasets with the R/Bioconductor package biomaRt. *Nat. Protoc.* **4**, 1184–91 (2009).

42. Sherry, S. T. dbSNP: the NCBI database of genetic variation. *Nucleic Acids Res.* **29**, 308–311 (2001).

43. Bycroft, C. *et al.* The UK Biobank resource with deep phenotyping and genomic data. *Nature* **562**, 203–209 (2018).

44. Purcell, S. *et al.* PLINK: A Tool Set for Whole-Genome Association and Population-Based Linkage Analyses. *Am. J. Hum. Genet.* **81**, 559–575 (2007).

45. Yang, J., Lee, S. H., Goddard, M. E. & Visscher, P. M. GCTA: A Tool for Genome-wide

Complex Trait Analysis. *Am. J. Hum. Genet.* **88**, 76–82 (2011).

46. Yang, J. *et al.* Conditional and joint multiple-SNP analysis of GWAS summary statistics identifies additional variants influencing complex traits. *Nat. Genet.* **44**, 369–375 (2012).

47. Giambartolomei, C. *et al.* Bayesian Test for Colocalisation between Pairs of Genetic Association Studies Using Summary Statistics. *PLoS Genet.* **10**, e1004383 (2014).

48. Bender Atik, R. *et al.* ESHRE guideline: recurrent pregnancy loss. *Hum. Reprod. Open* **2018**, (2018).

49. Definitions of infertility and recurrent pregnancy loss: A committee opinion. *Fertil. Steril.* **99**, 63 (2013).

50. Zegers-Hochschild, F. *et al.* The International Committee for Monitoring Assisted Reproductive. *Hum. Reprod.* **24**, 2683–2687 (2009).

51. Jauniaux, E., Farquharson, R. G., Christiansen, O. B. & Exalto, N. Evidence-based guidelines for the investigation and medical treatment of recurrent miscarriage. *Hum. Reprod.* **21**, 2216–22 (2006).

52. Recurrent Miscarriage, Investigation and Treatment of Couples (Green-top Guideline No. 17). Available at: https://www.rcog.org.uk/en/guidelines-research-services/guidelines/gtg17/. (Accessed: 2nd January 2020)

53. Magnus, M. C., Wilcox, A. J., Morken, N. H., Weinberg, C. R. & Håberg, S. E. Role of maternal age and pregnancy history in risk of miscarriage: Prospective register based study. *BMJ* **364**, (2019).

54. Ogasawara, M., Aoki, K., Okada, S. & Suzumori, K. Embryonic karyotype of abortuses in relation to the number of previous miscarriages. *Fertil. Steril.* **73**, 300–304 (2000).
