# Supplementary material for: The genetic architecture of sporadic and multiple consecutive miscarriage
Source: Nat Commun. 2020 Nov 25;11:5980. doi: 10.1038/s41467-020-19742-5 (PMC7689465; doi:10.1038/s41467-020-19742-5)
Supplement: Supplementary file 3 — Descriptions of Additional Supplementary Files [file 41467_2020_19742_MOESM3_ESM.pdf]

Descriptions of Additional Supplementary Files

Supplementary Data 1

Description: Descriptive statistics of cohorts in the sporadic and multiple consecutive miscarriage GWAS meta-analysis (.xlsx).

Supplementary Data 2

Description: Genotyping, quality control and association analysis details for participating cohorts (.xlsx).

Supplementary Data 3

Description: Genome-wide significant variants from miscarriage GWAS meta-analyses (.xlsx).

Supplementary Data 4

Description: Look-up of the markers previously associated with recurrent miscarriage (PMID: 27842992) in sporadic miscarriage (SM) EUR and multiple consecutive miscarriage (MCM) GWAS meta-analyses (.xlsx).

Supplementary Data 5

Description: Model fit and parameter estimates (with 95% confidence intervals) for heritability calculations (.xlsx).

Supplementary Data 6

Description: Results of the LD Score regression (LDSC) analysis for sporadic miscarriage EUR ancestry GWAS meta-analysis (.xlsx).

Supplementary Data 7

Description: ICD codes associated with sporadic miscarriage (.xlsx).

Supplementary Data 8

Description: ICD codes associated with multiple consecutive miscarriage (.xlsx).

Supplementary Data 9

Description: Mendelian randomization association of smoking, alcohol and body mass index with sporadic miscarriage (.xlsx).

Supplementary Data 10

Description: Top ten outcomes with the lowest p-values in the association with per MCM allele association and p-value (.xlsx).

Supplementary Data 11

Description: Results of the FUMA candidate SNP mapping for SM European ancestry meta-analysis (.xlsx).

Supplementary Data 12

Description: Results of the FUMA candidate gene mapping for SM European ancestry meta-analysis (.xlsx).

Supplementary Data 13

Description: Results of the FUMA candidate SNP mapping for MCM meta-analysis (.xlsx).

Supplementary Data 14

Description: Results of the FUMA candidate gene mapping for MCM meta-analysis (.xlsx).

Supplementary Data 15

Description: Selection of most likely candidate gene for each region based on multiple data sources (.xlsx).
